# Exploring the Impact of Dopaminergic Treatment on Creative Expression in Parkinson’s Disease: A Scoping Review

**DOI:** 10.3390/jcm14124119

**Published:** 2025-06-10

**Authors:** Giulia Marafioti, Laura Culicetto, Carla Susinna, Giuseppe Di Lorenzo, Angelo Quartarone, Viviana Lo Buono

**Affiliations:** IRCCS Centro Neurolesi “Bonino-Pulejo”, S.S. 113 Via Palermo C. da Casazza, 98124 Messina, Italy; giulia.marafioti@irccsme.it (G.M.); carla.susinna@irccsme.it (C.S.); giuseppe.dilorenzo@irccsme.it (G.D.L.); angelo.quartarone@irccsme.it (A.Q.); viviana.lobuono@irccsme.it (V.L.B.)

**Keywords:** creativity, divergent thinking, dopaminergic treatment, Parkinson’s

## Abstract

**Background/Objectives**: Creativity is a complex cognitive process influenced by multiple factors, including neurobiological mechanisms and neurotransmitter regulation. In Parkinson’s disease (PD), dopaminergic therapy has been associated with both increases and decreases in creative output, raising questions about its underlying mechanisms and clinical relevance. **Methods**: A scoping review was conducted following the PRISMA guidelines to evaluate studies assessing the effects of dopaminergic therapy on creativity in PD patients. The search was performed in January 2025 across PubMed, Scopus, Web of Science, and Embase databases using predefined search terms. **Results**: Seven studies met the inclusion criteria, reporting mixed outcomes. Some found enhanced artistic expression and divergent thinking in patients treated with dopamine agonists, while others observed no significant change. Increased creativity appeared more common in individuals with pre-existing artistic tendencies rather than as a new emergence due to medication. **Conclusions**: Dopaminergic therapy may amplify pre-existing creative inclinations in PD patients but does not consistently induce creativity across individuals.

## 1. Introduction

Parkinson’s disease (PD) is a progressive neurodegenerative disorder primarily affecting the dopaminergic neurons of the nigrostriatal pathway [1], which connects the pars compacta of the substantia nigra to the dorsal striatum (putamen and caudate nucleus) [1]. The loss of dopamine in this neural circuit impairs motor system functionality, leading to characteristic symptoms such as tremors and bradykinesia [2]. In addition to dopaminergic degeneration, Parkinson’s disease has been associated with persistent neuroinflammation. Studies have shown activation of microglia and increased levels of pro-inflammatory cytokines, such as TNF-α and IL-1β. There is also evidence of complement system activation in affected brain regions [3]. These findings suggest that inflammation contributes to neuronal loss and disease progression [4].

More recently, alterations in the orexin system have also been implicated. Orexin is a neuropeptide produced in the hypothalamus and is involved in arousal, sleep, and neuroprotection. Reduced orexin levels have been linked to several non-motor symptoms in PD, including sleep disturbances and fatigue. Its dysregulation may reflect broader hypothalamic involvement in the disease process [4]. However, PD extends far beyond motor dysfunction. A wide range of non-motor symptoms, including autonomic dysfunction, olfactory impairment, cognitive deficits, mood disturbances, and sleep alterations are now recognized as core features of the disease and significantly impact patients’ quality of life [5,6,7]. Dopaminergic therapy, which remains the cornerstone of symptom management in PD, includes different pharmacological approaches. The most used is Levodopa (L Dopa), a dopamine precursor that is converted to dopamine in the brain to broadly restore dopaminergic tone—a strategy referred to as dopamine replacement therapy. Dopamine agonists (DAs), on the other hand, directly stimulate dopamine receptors, particularly the D2 and D3 subtypes, without requiring metabolic conversion. Due to their more selective action on mesolimbic pathways, DAs are more frequently associated with neuropsychiatric effects, including impulsivity and enhanced creativity [8,9]. Both types of treatment are known to influence not only motor symptoms but also cognitive and emotional processes, such as reward, motivation, and creative thinking [10]. Research suggests that dopaminergic stimulation, particularly through dopamine replacement therapy or dopamine receptor agonists such as pramipexole or ropinirole, can affect brain regions involved in creativity, such as the prefrontal cortex, temporal lobes, and basal ganglia. Remarkably, some individuals with PD experience a sudden burst of creativity following dopamine therapy, often leading them to produce visual art, sculpture, literature, and poetry [11].

Creativity is widely recognized as a complex, multifaceted phenomenon that resists a singular definition. It is often described as the ability to generate ideas, solutions, or expressions that are not only novel but also appropriate and valuable within a specific context [12,13]. Contemporary theories highlight their foundation in both cognitive and emotional processes, with divergent thinking, originality, and problem-solving capacities interacting with personal, social, and environmental factors [14]. Additionally, creativity is considered a dynamic process, involving not only the generation of new ideas but also the evaluation and refinement of those ideas to ensure their utility and impact [15].

Creativity in PD seems to be linked to the intricate interplay between dopaminergic systems and other brain regions, including the prefrontal cortex, the temporal lobes, and the ventral striatum, which are involved in higher-order cognitive functions [16,17]. Dopaminergic modulation of these systems may lead to altered states of cognitive flexibility and reward processing, thereby facilitating the emergence of creative behaviors. In particular, the prefrontal cortex, which is responsible for executive functions such as planning, decision-making, and problem-solving, may become more flexible under dopaminergic stimulation, thereby enhancing creativity [16,18,19]. In contrast, the role of the mesolimbic system, which is responsible for reward processing and motivation, is also critical. Dopamine’s impact on the mesolimbic system can heighten sensitivity to rewarding stimuli, potentially driving a heightened focus on creative activities. However, this same increased dopaminergic activity can also contribute to impulsive or compulsive behaviors, such as hypersexuality or gambling, suggesting that the creative behaviors may be a side effect of overstimulation of reward pathways [20,21]. While increased creative expression has been observed in some PD patients receiving these medications, emerging evidence suggests that this enhancement may overlap with impulsive behaviors [22]. Some studies propose that the impulsive tendencies associated with impulse control disorders (ICDs) could contribute to excessive or compulsive creative output, making it challenging to differentiate between true creativity and compulsive behaviors [23,24]. The exact mechanisms by which PD may be related to an increase in artistic expression remain unknown. Engaging in creative activities can significantly enhance the quality of life (QoL) for individuals with PD, allowing them to express their emotions, connect with others, and preserve a sense of purpose and personal identity. Furthermore, certain creative pursuits have been shown to improve visual–cognitive skills, hand dexterity, general motor function, and other related abilities. However, an excessive focus on creative endeavors can sometimes lead to the neglect of essential daily activities such as social interactions, household chores, and personal care. This imbalance can negatively affect the overall well-being of PD, as their creative pursuits may become so consuming that they interfere with other important aspects of their lives. This scoping review aims to examine the existing literature on the topic, identifying key findings and gaps in understanding the interplay between dopaminergic therapy and creativity in individuals with PD, with particular attention to the clinical relevance of these findings for treatment optimization and patient quality of life.

## 2. Materials and Methods

**Figure 1:**
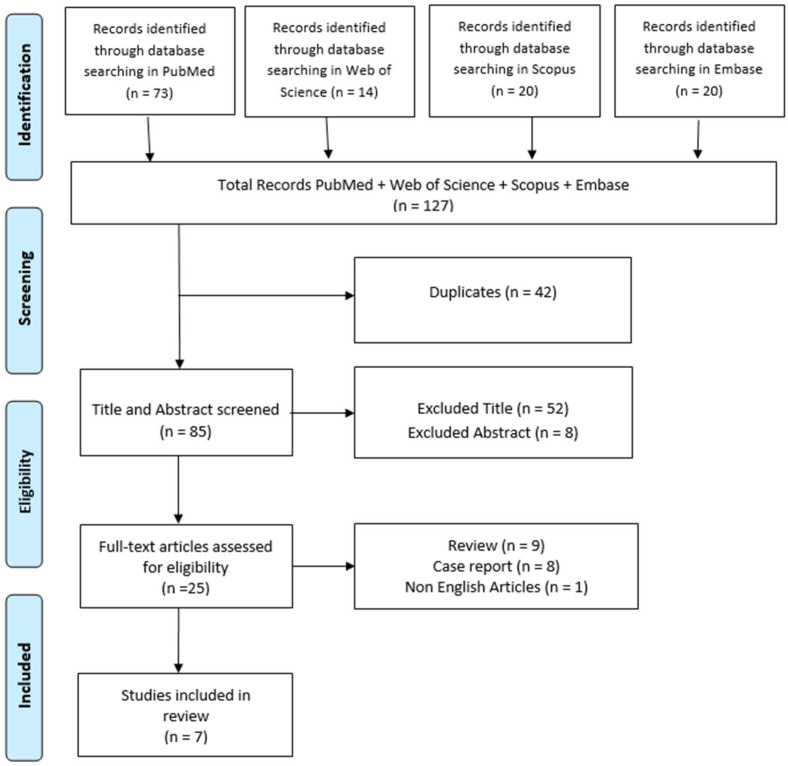
Search and selection of eligible articles.

A scoping review was conducted to examine the effects of dopaminergic therapy on creativity in people with PD. This review adhered to the PRISMA extension for scoping reviews (PRISMA-ScR) guidelines to ensure methodological rigor [25] (Figure 1) and was registered on OSF https://doi.org/10.17605/OSF.IO/YWCJV on date 25 March 2025.

### 2.1. PCC Model

The PCC framework (Population, Concept, and Context), as recommended by Pollock et al. [26], was used to construct clear and meaningful objectives and eligibility criteria for this scoping review. The population of interest consists of people with PD who exhibit creative behaviors. The central concept pertains to dopaminergic treatment, while the context encompasses studies conducted in various healthcare and rehabilitative settings, without geographic or cultural restrictions.

### 2.2. Search Strategy

A scoping review of currently published studies was performed in January 2025 using the following databases: Scopus, PubMed, Web of Science, and Embase. The search combined the following terms: (“creative” [All Fields] OR “creatively” [All Fields] OR “creatives” [All Fields] OR “creativities” [All Fields] OR “creativity” [MeSH Terms] OR “creativity” [All Fields] OR “creativeness” [All Fields]) AND ((“dopamine agonists” [Pharmacological Action] OR “dopamine agonists” [MeSH Terms] OR (“dopamine” [All Fields] AND “agonists” [All Fields]) OR “dopamine agonists” [All Fields] OR “dopaminergics” [All Fields] OR “dopamine” [MeSH Terms] OR “dopamine” [All Fields] OR “dopaminergic” [All Fields] OR “dopaminergically” [All Fields]) AND (“therapeutics” [MeSH Terms] OR “therapeutics” [All Fields] OR “treatments” [All Fields] OR “therapy” [MeSH Subheading] OR “therapy” [All Fields] OR “treatment” [All Fields] OR “treatment s” [All Fields])) AND (“parkinson disease” [MeSH Terms] OR (“parkinson” [All Fields] AND “disease” [All Fields]) OR “parkinson disease” [All Fields] OR “parkinson s” [All Fields] OR “parkinsons” [All Fields] OR “parkinson” [All Fields] OR “parkinsonian disorders” [MeSH Terms] OR (“parkinsonian” [All Fields] AND “disorders” [All Fields]) OR “parkinsonian disorders” [All Fields] OR “parkinsonism” [All Fields] OR “parkinsonisms” [All Fields] OR “parkinsons s” [All Fields]). Although the search strategy included a broad set of terms related to “creativity”, studies that described specific forms of artistic expression (e.g., painting, music, literature) without explicitly referring to creativity may not have been captured. This limitation should be considered when interpreting the comprehensiveness of the included literature. No restriction was placed on the publication year of the article.

### 2.3. Inclusion and Exclusion Criteria

The inclusion criteria were as follows: (i) subjects with PD and creative behaviors; (ii) studies describing pharmacological treatments for the development of creativity in PD; (iii) studies assessing creativity using neuropsychological tests or clinical interviews; (iv) studies exploring the impact of dopaminergic treatment in PD; (v) articles published in English; (vi) studies conducted in healthcare or rehabilitation settings, with no geographical or cultural restrictions. The following were excluded: (i) conference proceedings or commentaries; (ii) reviews; (iii) letters to the editor; and (iv) grey literature, such as conference proceedings, reports, and non-peer-reviewed literature. This exclusion was made to ensure methodological rigor by focusing on peer-reviewed articles, which are considered to provide a higher level of quality and reliability.

### 2.4. Study Selection

To minimize bias and ensure a rigorous selection process, two authors (G.M. and L.C.) independently screened the titles and abstracts of the studies based on the inclusion and exclusion criteria. Any discrepancies were resolved through collaborative discussion, with consultation from a third author (V.L.B). This multi-step approach ensured that at least three researchers independently assessed each article. The final inclusion or exclusion decision was based on predefined criteria regarding the population (e.g., PD patients with creative behaviors), the concept (e.g., dopaminergic therapy), and the context (e.g., healthcare or rehabilitation settings). In cases of persistent disagreement, the final decision involved all authors.

### 2.5. Data Extraction and Analysis

The studies that met the inclusion criteria were summarized based on the following points: (1) study characteristics, including the type of study and the country where the data were collected; (2) patient characteristics, such as the sample size, age, gender, duration of disease, and the level of education; and (3) key findings and relevant outcomes. Following the full-text selection, data were extracted from the included studies and reported in a table using Microsoft Excel (Version 2021). Moreover, the agreement between the two reviewers (G.M. and L.C.) was assessed using the kappa statistic. The kappa score, with an accepted threshold for substantial agreement set at >0.61, was interpreted to reflect substantial concordance between the reviewers. This criterion ensures a robust evaluation of the inter-rater reliability, ensuring a high level of agreement in the data extraction process.

## 3. Results

The seven studies reviewed explore the relationship between dopaminergic therapy and creativity in subjects with PD (Table 1). The findings suggest that dopamine plays a role in creative expression; however, the relationship is complex and appears to be influenced by multiple factors, including medication dosage, impulsivity, and cognitive flexibility. Some studies indicate that DAs enhance creativity, while others suggest that this phenomenon may not be a direct consequence of medication alone.

### 3.1. Dopamine Agonists and Enhanced Creativity

Three studies support the idea that dopaminergic therapy, particularly dopamine agonists, is associated with increased creative output in PD. Garcia-Ruiz et al. [27] identified a group of PD subjects who developed artistic abilities after starting DA treatment, with painting being the most common creative activity. Similarly, Lhommée et al. [28] observed that creativity was linked to DA therapy but diminished after deep brain stimulation (DBS). This decline coincided with a marked reduction in dopaminergic medication following surgery, suggesting that the decrease in dopamine levels may contribute to the reduction in creative output. These findings reinforce the role of dopamine in sustaining creative behavior, particularly in PD subjects.

The study by Faust-Socher et al. [24] further reinforces this hypothesis, demonstrating that PD subjects on dopaminergic therapy performed better than healthy controls in divergent thinking tasks, particularly in tasks requiring flexibility and originality. This enhancement was not associated with ICDs, suggesting that creativity in PD may emerge independently of the compulsive behaviors often linked to DA.

### 3.2. Creativity as a Positive Side Effect of Dopamine Therapy

The study by Pérez-Torre [29] explores the role of dopaminergic therapy in promoting creative expression. In a small observational case series involving six individuals with Parkinson’s disease, one patient treated with dopamine agonist monotherapy (ropinirole) produced notably more imaginative and intricate drawings compared to the five others receiving only levodopa. Although based on a single case, this observation has generated interest regarding the potential influence of dopamine agonists on creative expression in PD. This case study suggests that dopamine-induced creativity may be an individualized phenomenon, influenced by personal predispositions and underlying neurobiological factors. Conversely, Canesi et al. [30] challenge the notion that dopaminergic therapy directly induces creativity. Their study compared professional artists with and without PD, as well as PD subjects who developed artistic tendencies post-diagnosis. The authors found that creativity scores were higher in professional artists regardless of PD status, and there was no correlation between creativity and DA dosage. This suggests that dopaminergic therapy may act as a facilitator rather than a generator of creativity, amplifying existing artistic inclinations rather than creating them de novo.

### 3.3. Limitations of Dopaminergic Therapy in Enhancing Creativity

Despite the evidence supporting dopamine’s role in creativity, some studies present contrasting results. Salvi et al. [31] found no strong evidence that dopamine replacement therapy improved creative thinking. Instead, PD subjects on medication exhibited reduced cognitive flexibility and performed worse on insight-based problem-solving tasks. This suggests that while dopamine may enhance spontaneous artistic output, it does not necessarily improve structured creative cognition. Likewise, Heldmann et al. [32] found that PD subjects showed reduced originality in divergent thinking tasks compared to healthy controls, despite being on dopaminergic medication. These findings imply that the relationship between dopamine and creativity is not straightforward. While some PD subjects may experience bursts of artistic expression, dopaminergic therapy does not universally enhance all aspects of creative cognition.

**Table 1:** Characteristics of the studies included.

Authors	Sample Size	Disease Duration	Cognitive, Emotional, and Motor Assessment	Creativity Assessment	Methods/Treatments	Outcomes
Canesi et al., 2015 [30]	PD group creative: F/M 5/7, age 54.9 (11.4) PD group artists: F/M 5/7, age 53.7 ± 10.4 PD group non-artists: F/M 5/7, age 57.5 ± 9.2 HC artist: F/M 5/7, age 56.9 ± 10.7 HC non-artist: F/M 5/7, age 53.4 ± 8.8	PD group creative: 7.0 (3.3) years PD group artists: 7.1 (3.4) years PD group non-artists: 7.1 (3.8) years	UPDRS-III MMSE FAB GDS HAM-A	ATTA mMIDI	Comparison of professional and non-professional artists with PD and healthy controls using creativity tests	Creativity scores higher in professional artists, not correlated with dopaminergic therapy dosage
Faust-Socher et al., 2014 [24]	PD group 27: F/M 11/17, age 62 ± 7 HC group 27: F/M 16/12 age 59 ± 9	PD group: 5.8 63.9 years	MoCA	TACT RAT	27 PD patients on dopamine agonists and/or levodopa compared to 27 healthy controls in creativity tasks	PD patients performed better than controls in divergent thinking tasks; unrelated to impulse control disorder
Garcia-Ruiz et al., 2019 [22]	21 PD patients (20 PD, 1 restless legs syndrome)	NA	NA	Ardouin scale for creativity assessment	21 PD patients treated with dopamine agonists; analyzed their artistic tendencies post-treatment	Most patients exhibited new artistic abilities post-dopaminergic treatment, mainly painting
Heldmann et al., 2024 [32]	PD group 20: F/M 10/10, age 67.3 ± 7.3 HC group 20: F/M 11/10, age 68.1 ± 9.9	PD group: 7.15 (5.5) years	UPDRS-III PANDA BDI-II	AUT RAT	20 PD patients and 20 healthy controls assessed on divergent/convergent thinking and cognitive estimation	No major differences in convergent thinking; PD patients had reduced originality in divergent thinking
Lhommée et al., 2014 [28]	PD group creative 11: F 45.5%, age 53 HC group non-creative 22: F 31.8%, age 56.5	PD group creative: 11(9;12) years	UPDRS-III MDRS WCST BDI-II SAS Ardouin scale	TACT RAT	11 creative PD patients undergoing DBS were compared to 22 non-creative PD patients pre- and post-surgery	Creativity was linked to dopamine agonists but diminished after DBS due to reduced medication
Pérez-Torre et al., 2021 [29]	PD group: 6 M, age 70 ± 5	PD group: 5.5 ± 2.0 years	NA	Spontaneous drawing assessment	6 PD patients asked to draw spontaneously for 2 min; one was taking a dopamine agonist.	Patient on dopamine agonist showed significantly more detailed and creative drawings
Salvi et al., 2021 [31]	PD group: F 4, age 56.5 ± 9 HC group: 26 F, 15 age 61.3 ± 7	NA	MMSE FAB RPM	Rebus Puzzles AUT CRA HABPS QUIP-ICD and RS BIS-11A	PD patients tested ‘on’ and ‘off’ medication in various creative problem-solving tasks	DRT did not enhance creativity; patients showed reduced flexibility and made more errors in tasks

Legend: Not available (NA); female (F); male (M); healthy controls (HC); Unified Parkinson’s Disease Rating Scale–motorscore (UPDRS-III); Mini-Mental State Examination (MMSE); Raven’s Progressive Matrices (RPM); Frontal Assessment Battery (FAB); Geriatric Depression Scale (GDS), Wisconsin Card Sorting Test (WCST); Mattis Dementia Rating Scale (MDRS); Hamilton Anxiety Scale (HAM-A); Abbreviated Torrance Test for Adults (ATTA); Minnesota Impulsive Disorders Interview (mMIDI); Montreal Cognitive Assessment (MocA); Tel Aviv Creativity Test (TACT); Alternative Uses Task (AUT); Compound Remote Associates (CRA); Barratt Impulsiveness Scale (BIS-11A); QUIP-Rating Scale (QUIP-ICD and RS); Hobbyism and Artistic-like Behaviors Punding Scale (HABPS); Remote Associates Task (RAT), Starkstein Apathy Scale (SAS); dopamine replacement therapy (DRT).

## 4. Discussion

The relationship between dopaminergic therapy and creativity in PD appears to be complex and influenced by multiple factors. The findings from the reviewed studies present contrasting perspectives on the role of dopamine in creativity. Several studies have indicated an association between dopaminergic therapy, particularly dopamine agonists, and increased creative output in PD people. While levodopa is crucial for maintaining overall dopaminergic tone in the brain, it is less frequently implicated in triggering creative behavior [28]. In contrast, DAs have been more consistently associated with stimulating creativity, likely due to their more direct influence on specific dopaminergic pathways that are thought to be involved in creative processes.

Research suggests that, similar to other behavioral changes, dopamine agonists play a pivotal role in fostering creative behavior. Creativity, inherently linked to the brain’s reward system, thrives on the ability to form novel associations and ideas. Dopamine D2–D3 agonists, which have a high affinity for D3 receptors predominantly located within the mesolimbic pathway, may enhance this process by facilitating the flow of creative thoughts and their expression [33]. By modulating dopaminergic activity in this key brain region, these agonists likely promote greater cognitive flexibility, enabling free associations and more dynamic artistic production. This heightened capacity for associative thinking may be crucial in driving the generation of innovative ideas and the manifestation of creative output. For instance, some reports describe the emergence of artistic abilities, such as painting, following the initiation of dopaminergic treatment [34]. In addition, a decline in creative expression has been observed following DBS, likely resulting from a reduction in dopaminergic medication, emphasizing the critical role of dopamine in sustaining creative behavior in PD [28]. A case study further supports this hypothesis, where subjects undergoing dopamine agonist treatment exhibited highly creative and detailed drawings, suggesting that dopamine-related creativity may be influenced by neurobiological factors and individual predispositions [29].

Two key mechanisms have been proposed to explain dopamine’s role in creativity. The first is latent inhibition (LI), the brain’s ability to filter out irrelevant stimuli. For example, reduced LI, often observed in psychosis, appears to improve divergent thinking by expanding associative networks, leading to greater creative output. The literature suggests that lower LI, especially in individuals with high IQ, is linked with greater lifetime creative achievements. This process is believed to be influenced by dopamine levels acting through the nucleus accumbens [35,36]. The second mechanism involves novelty-seeking behavior, as creative individuals tend to seek new experiences. This trait has been associated with brain regions containing or receiving dopaminergic input, including the ventral striatum, substantia nigra, ventral tegmental area, and hippocampus. Although there has been debate over whether novelty-seeking is exclusive to PD subjects with impulse control disorders (ICDs), some studies suggest that creativity can emerge independently of compulsive or obsessive behaviors [37]. However, some studies question the idea that dopaminergic therapy directly induces creativity, suggesting instead that it may act more as a facilitator—amplifying pre-existing creative tendencies—rather than as a direct generator of creativity [30]. The discrepancies in findings across different studies may also be attributed to the diversity of assessment tools used to measure creativity. For example, the Guilford Alternate Uses Task (AUT) is commonly used to evaluate divergent thinking, which is one aspect of creativity, while other assessments focus on fundamental cognitive processes related to creative thinking. Additionally, tests like the Abbreviated Torrance Test for Adults and the Remote Association Task (RAT) are designed to measure different facets of creativity, such as the ability to generate novel ideas or to recognize connections between seemingly unrelated concepts. The variation in results could stem from these tools assessing distinct dimensions of creativity, each emphasizing different cognitive skills and processes. As a result, the way creativity is measured in research can significantly impact the outcomes and interpretations of studies in this area.

Regarding gender, no significant differences have been reported in the occurrence of creative behaviors, suggesting that this phenomenon is not influenced by sex. In addition, only one of the reviewed studies reported H&Y staging, and no clear correlation with creativity was described. Therefore, no firm conclusions can be drawn regarding the relationship between disease severity and creative expression. While current evidence does not systematically examine the role of PD subtypes in creative expression, it is plausible that different clinical phenotypes—such as tremor-dominant versus akinetic-rigid forms, or subtypes marked by prominent mood or cognitive features—may differentially respond to dopaminergic stimulation. These differences could potentially influence both the emergence and nature of creative behaviors. Future research should stratify patients by clinical subtype to clarify whether certain forms of PD are more likely to exhibit enhanced creativity with dopaminergic therapy. Such insights could inform more individualized treatment strategies and behavioral expectations.

Creativity is considered to be one of the most important characteristics that humans possess. Similar to other higher mental functions, it is assumed that creativity emerges from fundamental cognitive operations, including working memory, sustained attention, planning, cognitive flexibility, mentalizing, and abstraction [38]. These cognitive functions are commonly impaired in PD; however, the altered dopaminergic system, particularly with medication, may paradoxically facilitate creative processes by promoting a more flexible, less inhibited cognitive style. In conclusion, dopaminergic therapy, particularly DAs, appears to facilitate creativity in PD subjects, but it is not consistently associated with measurable improvements in creative ability. It is likely that such therapy acts as a catalyst for the expression of pre-existing artistic tendencies rather than as an inducer of creativity. Further research integrating neurobiological and psychometric approaches will be essential to elucidate the mechanisms underlying this phenomenon and its impact on patient QoL. These findings, beyond their theoretical implications, may also bear clinical relevance. Understanding how dopaminergic therapy modulates creativity could help inform more personalized care strategies, fostering well-being and quality of life through meaningful engagement in creative activities, especially for individuals with pre-existing inclinations.

### Limitations and Future Directions

The studies included in this review present several limitations that should be addressed in future research. First, the majority of included studies had small sample sizes, significantly limiting the generalizability and statistical power of the results. As a consequence, observed associations between dopaminergic therapy and creativity in PD may be driven by individual variability rather than robust trends. Second, there is a lack of standardization in the tools used to assess both creativity and cognitive function. The included studies employ different tools and approaches, making direct comparisons difficult and leading to inconsistent interpretations. Establishing standardized methodologies for evaluating creative thinking, cognitive flexibility, and problem-solving abilities would enhance comparability and reliability across studies. Additionally, while cognitive function has been assessed in several studies, only two studies [28,30] specifically examined emotional factors, such as apathy and depression. Given the strong interplay between mood, motivation, and creativity, future research should integrate comprehensive assessments of emotional well-being to determine how these factors influence creative expression in PD patients. A wide range of cognitive assessment tools have been used, including the Mattis Dementia Rating Scale (MDRS), Montreal Cognitive Assessment (MoCA), and Mini-Mental State Examination (MMSE), making it difficult to compare results across studies. Moreover, only Canesi et al. [30] included an anxiety scale, further underscoring the need for a more comprehensive assessment of emotional influences on creativity. The lack of standardized neuropsychological and emotional assessments across studies remains a major challenge in drawing definitive conclusions regarding the role of dopaminergic therapy in creativity. To address these gaps, future research should adopt a more unified methodological approach, incorporating comprehensive cognitive and emotional assessments. Neuroimaging techniques, such as functional magnetic resonance imaging (fMRI) and positron emission tomography (PET), could provide valuable insights into how dopamine modulates brain networks involved in creative cognition and whether these effects are transient or sustained over time. Recent evidence suggests that positron emission tomography (PET), traditionally used for early PD diagnosis, may also represent a cost-effective tool for exploring neural substrates underlying creative processes [39]. Investigating the relationship between dopamine, cognitive flexibility, and creativity at different stages of PD may also help clarify whether creativity is primarily influenced by disease severity, medication dosage, or individual predispositions. Finally, while some studies link enhanced creativity with ICDs, others report creative expression independent of compulsive behaviors. This inconsistency raises the question of whether increased creativity is a direct pharmacological effect or a manifestation of broader changes in behavioral regulation. Without longitudinal data and comprehensive behavioral assessments, this question remains unresolved. Future research should explore whether increased artistic expression in PD patients is a direct effect of dopaminergic therapy or a byproduct of broader changes in behavioral regulation. Understanding these mechanisms is essential for optimizing treatment strategies that balance symptom relief with the minimization of cognitive and behavioral side effects. Finally, a more individualized approach is needed in future research. Considering genetic, neurobiological, and psychological predispositions could help identify which patients are most likely to experience enhanced creativity in response to dopaminergic therapy. Longitudinal studies with larger cohorts and multidimensional assessments of creativity, cognition, and emotional well-being would provide a more comprehensive understanding of this phenomenon and its potential clinical significance. Future research should adopt larger sample sizes, unified methodological frameworks, and multidimensional assessment protocols encompassing cognitive, emotional, and behavioral domains. It will also be essential to explore genetic, psychological, and neurobiological predispositions to identify which PD patients are more likely to exhibit creativity in response to treatment. Only through such an integrated and critical approach can we gain a clearer understanding of this intriguing and clinically relevant phenomenon.

## 5. Conclusions

Creativity is a complex trait and any attempt to define or measure creativity is bound to be incomplete. The reviewed studies collectively highlight the complex interplay between dopamine and creativity in PD. While there is evidence suggesting that dopamine agonists can enhance artistic expression and divergent thinking, creativity does not appear to be a direct consequence of dopaminergic therapy alone. Instead, the effects seem to be moderated by individual predispositions, cognitive flexibility, and impulse control mechanisms. The discrepancy in findings suggests that further research is necessary to determine the precise neural mechanisms underlying this phenomenon. Future studies should consider larger sample sizes, longitudinal designs, and the inclusion of pre-morbid creative tendencies to better understand whether dopamine acts as a catalyst or merely an enabler of creativity in PD. Additionally, investigating the differences between spontaneous artistic production and structured problem-solving tasks may provide further clarity on how dopaminergic therapy influences various dimensions of creativity.

## Data Availability

Not applicable.
